# Temporal oscillations in preference strength provide evidence for an open system model of constructed preference

**DOI:** 10.1038/s41598-021-87659-0

**Published:** 2021-04-14

**Authors:** Peter D. Kvam, Jerome R. Busemeyer, Timothy J. Pleskac

**Affiliations:** 1 University of Florida, Gainesville, USA; 2 Indiana University, Bloomington, USA; 3 University of Kansas, Lawrence, USA

**Keywords:** Psychology, Human behaviour

## Abstract

The decision process is often conceptualized as a constructive process in which a decision maker accumulates information to form preferences about the choice options and ultimately make a response. Here we examine how these constructive processes unfold by tracking dynamic changes in preference strength. Across two experiments, we observed that mean preference strength systematically oscillated over time and found that eliciting a choice early in time strongly affected the pattern of preference oscillation later in time. Preferences following choices oscillated between being stronger than those without prior choice and being weaker than those without choice. To account for these phenomena, we develop an open system dynamic model which merges the dynamics of Markov random walk processes with those of quantum walk processes. This model incorporates two sources of uncertainty: epistemic uncertainty about what preference state a decision maker has at a particular point in time; and ontic uncertainty about what decision or judgment will be observed when a person has some preference state. Representing these two sources of uncertainty allows the model to account for the oscillations in preference as well as the effect of choice on preference formation.

## Introduction

Contemporary theories of decision making propose that people form preferences as they accumulate information about the different outcomes they can obtain^1^. In this way, preferences change over time as value is dynamically constructed from the attributes of the available choice options^2–7^. These accounts also suggest that, although attention may fluctuate between attributes so that preferences change stochastically over time^3,8,9^, a person’s preference between a pair of choice alternatives should gradually accumulate toward a “favored” option. For example, when making a choice between restaurants a person’s attention might fluctuate between considering quality, cost, and convenience across time, but these evaluations will eventually lead them to favor one of the possible options. In most cases, the average trajectory of preference in a binary choice should thus appear as a gradual and monotonic increase toward one option over the other.

Models of preference based on Markov random walks or diffusion processes generally predict this monotonic average trajectory^10^, but non-classical accounts based on quantum walks^11–14^ are at odds with this account. Instead, quantum walk models make the divergent prediction that preference should oscillate over time, systematically going back and forth between favoring one option and another as preferences evolve. The difference in predictions can be understood by drawing an analogy between preferences moving over a bounded preference scale driven by attribute information and either sand or water moving within a bounded region blown by wind. In the Markov account, wind (new information) pushes sand (a probability distribution) toward one edge (end of the preference scale) where it piles up, reaching a stable equilibrium. In the quantum account, the wind pushes a wave of water (amplitude distribution) toward one edge, but it reverberates back off the edge until the wind blows it forward again, making the wave slosh back and forth over time.

Fundamentally, these diverging predictions arise from differences in how these models represent uncertainty in preferences. Classical dynamic models, as well as heuristic accounts^15,16^ and many other theories of preferential choice^17^, suggest that a definite state of preference exists at each moment in time. This implies that at any given point in time, a person holds a concrete valuation for each alternative and that the concrete valuations carve out a definite trajectory as they change over time. Uncertainty about preferences in these models is entirely *epistemic*, capturing an outside observer’s ignorance about what the decision maker thinks and feels internally at any moment. Conversely, quantum models incorporate *ontic uncertainty*, which describes a person’s internal uncertainty about different responses (choices, judgments, ratings) that could be realized at a specific moment^18–22^.

Note that the distinction between epistemic and ontic uncertainty is different than the distinction between epistemic and aleatory uncertainty. Epistemic and aleatory uncertainty refer to a classical system where they can be made arbitrarily small with more precise measurements or a more detailed theoretical description of the actual system^20,23^. Ontic uncertainty becomes relevant when a quantum system is considered, where we can know exactly the superposition state of the system and yet simultaneously different events still have a nonzero potential to be realized. As a result, ontic uncertainty can be reduced by eliciting a response. By forcing a decision maker to choose between two options, they must “collapse” on preferences consistent with one of the choice options. This resolution of uncertainty via a response has important consequences—it can create sequential effects where responses made in sequence affect one another by altering a decision maker’s cognitive state when a response is elicited^12–14^. Therefore, adding quantum dynamical elements to a model should not only result in oscillations in mean preference, but also diverging preference evolution after a decision maker is forced to choose between options (as opposed to conditions not requiring a initial choice)^12^.

Naturally, a complete account of how people consider their options entails both types of uncertainty: epistemic uncertainty regarding what an outside observer knows about how individuals will react to the choice options they are offered, and ontic uncertainty within the decision maker about what states of preference they experience while deciding. In this article, we make the case for such a hybrid account of uncertainty during the decision process, connecting well-established and new empirical work on the evolution of preference to a new model that incorporates both epistemic and ontic uncertainty. We refer to this as an *open system* model^24,25^. This model incorporates classical Markov random walk properties to describe epistemic uncertainty as well as a non-classical quantum walk properties that describe how a person’s preferences oscillate over time and how decisions reduce ontic uncertainty to affect subsequent responses.

**Figure 1:**
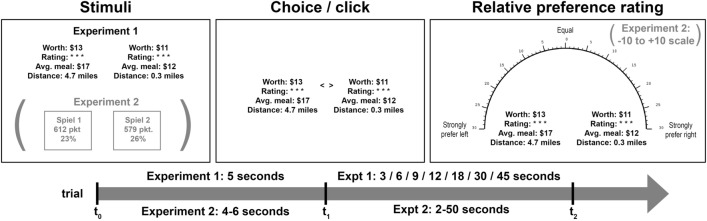
Outline of the structure of experiments 1 and 2.

To understand the pattern of average preference trajectories and how it ties into classical or quantum dynamics (or, as we suggest, a hybrid of the two), we conducted two studies. In these experiments, we traced out the preference trajectory that unfolds over time as participants considered a choice between (actual) restaurant gift cards (Experiment 1) or between monetary gambles (Experiment 2) (Fig. 1). The experiments are described in detail in the “Supplementary materials”. Both studies compared preference patterns following a choice between two options against preference patterns in absence of choice. Both studies were incentivized with real payoffs corresponding to the options participants were presented. The result from both experiments was that preference systematically oscillated over time, and that these oscillations depended on whether or not a choice was made prior to the preference rating. Such phenomena place strong constraints on dynamic theories of preference evolution and preferential choice, which we address after presenting the results.

## Results

For both experiments, we analyzed the *strength* of preference ratings, which was calculated as the absolute value of each rating (distance from the midpoint of the scale). This was done to match the choice and no-choice conditions, as there was generally no objectively better option and we cannot condition ratings on the chosen item in a no-choice condition. For Experiment 1, these strength of preference ratings ranged from 0 to 30 because the scale varied from $-30$ (strongly favor left) to $+30$ (strongly favor right). For Experiment 2, they ranged from 0 to 10 because the scale went from $-10$ (strongly favor left) to $+10$ (strongly favor right).

To analyze the results of these experiments, we carried out two main analyses on each experiment. The first was a polynomial regression model predicting mean strength of preference ratings as a quintic function of the time point at which they were drawn. There are several reasons for using a 5th order polynomial. Chiefly, it allows us to detect patterns of oscillation that involve at least four inversions (two cycles). A single cycle could be detected with a 3rd order polynomial, and we report this model for completeness (using a 3rd or 4th order polynomial still results in credible/significant effects), but the data appear to include at least two cycles. We are also limited to a quintic polynomial by the amount of data in the first experiment. With only six time points, a quintic polynomial is fully saturated and therefore testing its coefficients should identify any significant/credible effects in the data.

A key prediction of the quantum model is that making an initial choice should induce a change in the patterns of preference oscillation. Our previous work suggests that there should be some mean differences between the two conditions^12^, but the effect is also predicted to manifest in the *amplitude* of the oscillations between conditions at longer timescales^14^. Therefore, we also used the polynomial regression to identify differences between the choice and no-choice conditions comparing the 5th order coefficients between conditions.

A second prediction of the quantum and open system (with a partial quantum component) models that can be evaluated with the polynomial regression is that preference strength should oscillate over time. We fit the fifth order polynomial model to the choice and no-choice conditions separately, meaning that we can evaluate the higher-order (3+) coefficients within each condition to determine if there is credible oscillation, in addition to evaluating the differences between conditions.

The second analysis that we carry out for both experiments is a Gaussian process regression, which allows us to make inferences about *functions* that map time point onto mean preference strength as opposed to making inferences about the coefficients of a pre-specified function. This analysis is more general and in some ways more complete, as it considers all possible mappings between elicitation time and preference strength (within a set of weak assumptions) and assigns likelihoods to which mappings are most likely. This analysis allows us to construct a 95% highest density interval [HDI] on mean preference, spanning not just our data points but all possible time points that could be observed. By comparing differences in the HDI between choice and no-choice conditions, we can identify any instances where the two conditions diverge based on our data.

To summarize the main results of both experiments, we observed that preferences oscillated back and forth systematically over time. Furthermore, this oscillation depended on whether or not a choice was made prior to the preference rating. Choice apparently stabilized overall preferences so that they were more resistant to subsequent change, resulting in lower-amplitude oscillations in preference strength in the choice relative to no-choice conditions. As a consequence, we observed cases where preference strength was stronger in choice conditions than in no-choice conditions (which we refer to as *bolstering* effects) and cases where preference strength was weaker in choice conditions than in no-choice conditions (which we refer to as *suppression* effects).

### Experiment 1

**Figure 2:**
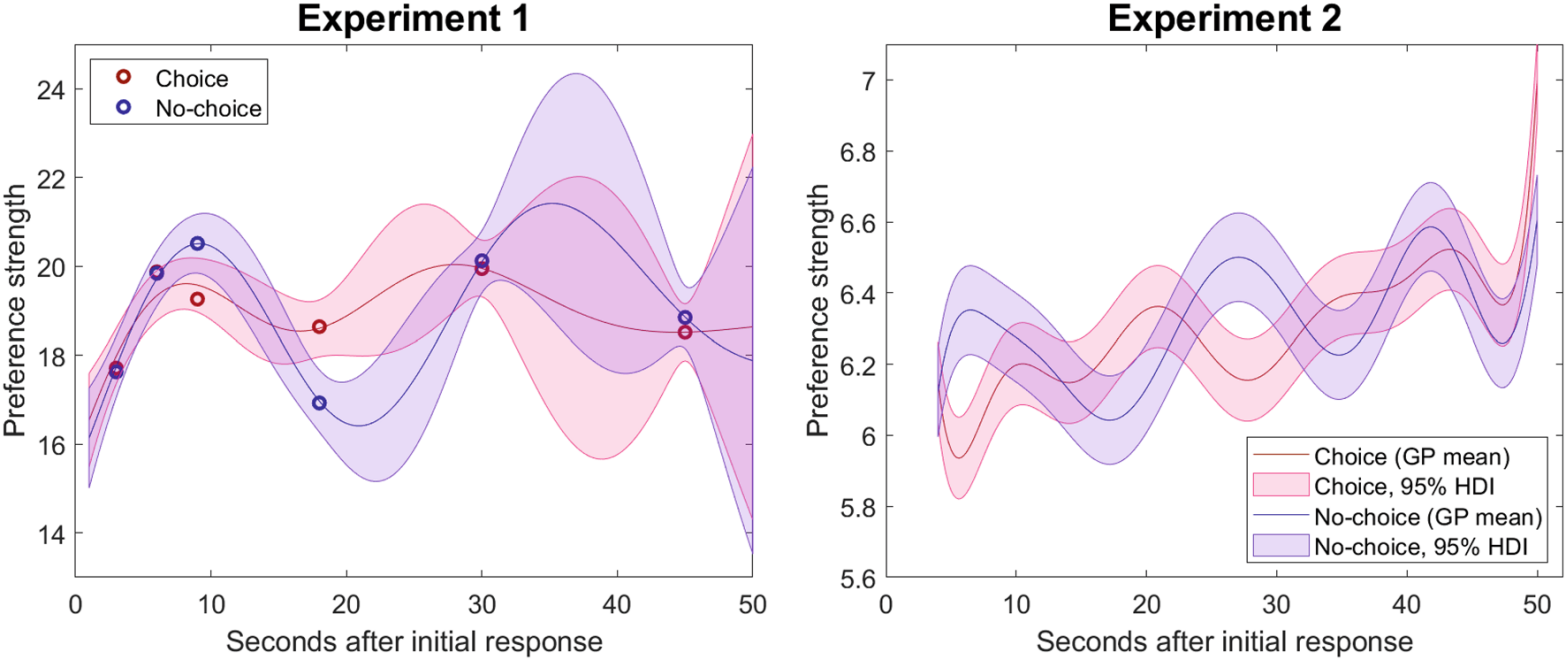
Plot of Gaussian process regression, which estimates the best functions mapping time point (x-axis) onto mean preference strength (y-axis). Filled areas indicate 95% most credible functions/error for choice (red) and no-choice (blue) conditions. Preference strength is on a 0–30 scale for Experiment 1 and 0–10 scale for Experiment 2.

Experiment 1 featured preference ratings between pairs of restaurant gift cards. These ratings could be elicited at any of six discrete time points: 3, 6, 9, 18, 30, or 45s following the initial responses. The mean preference ratings are shown as dark red (choice) and blue (no-choice) circles on the left side of Fig. 2. There were several notable characteristics of these ratings. First, when viewed across the sampled time points, the preference strength appeared to fluctuate back and forth over the 45-s span of delays in the experiment. Second, in the condition where participants had made a decision between the two options (choice), the ratings appeared to be more stable or show less fluctuations than those in the no-choice condition.

As suggested above, the first analysis we carried out examined the polynomial regression estimates to examine the average preference (intercept) and how it changed over time (1st+ order coefficients), including any oscillations in the data (3rd+ order coefficients) (Table 1). On average, mean preference strength was 17.06 on a 30-point scale in the no-choice condition, and 16.59 in the choice condition (credibly lower: $M_{diff}$ = 0.47, 95% HDI = [0.06, 0.83]). In both conditions, preference strength oscillated over time, evidenced by nonzero effects in the cubic, quartic, and quintic coefficients of the polynomial regression (last three rows of Table 1). This is true for both the choice condition and for the no-choice condition, indicating that there are credible oscillations within both conditions.

**Table 1:** Summary of the estimated coefficients (with 95% HDIs) of the polynomial for the choice and no-choice conditions, along with the estimated difference (95% HDI) between conditions for Experiment 1. Coefficients are standardized to make the scales comparable—raw coefficients can be obtained by dividing $b_n$ by the standard deviation of the time points for the corresponding term, $SD(t^ {\circ n})$ with $t = [3,6,9,12,18,30,45]$ (where $t^{\circ n}$ is element-wise power function, raising the values in *t* to the *n*th power) . Differences between choice and no-choice conditions that exclude zero are highlighted in bold.

Polynomial coefficient	No choice	Choice	Difference
Intercept ($b_0$)	17.06 [14.82, 19.04]	16.59 [14.72, 18.21]	**0.47 [0.06, 0.83]**
Slope ($b_1$)	9.33 [0.90, 18.05]	9.33 [2.71, 16.10]	$-0.00$ [$-1.95$, 1.96]
Quadratic ($b_2$)	$-39.90$ [$-67.24$, $-0.74$]	$-31.84$ [$-51.86$, $-7.39$]	$-7.98$ [$-15.88$, 2.22]
Cubic ($b_3$)	36.97 [10.08, 61.51]	23.53 [5.04, 40.67]	** 13.34 [4.81, 21.41]**
Quartic ($b_4$)	30.23 [3.64, 51.65]	27.80 [4.25, 46.80]	2.58 [$-0.61$, 4.86]
Quintic ($b_5$)	$-36.73$ [$-58.84, -8.51$]	$-28.90$ [$-47.44, -5.77$]	${\mathbf {-7.82}} {\mathbf {[-11.74, -2.54]}}$

In addition to the difference between the intercepts/mean preference strength in choice and no-choice conditions, there were also differences between these two conditions in the cubic and quintic components of the polynomial regression (standardized coefficients are shown in Table 1). These reflect the observation that the no-choice condition tended to show greater amplitude in its oscillations relative to the choice condition and are consistent with the interference effect reported for confidence in perceptual choice^12,13^.

As a result of these oscillations, there were both cases where preference in the choice condition was greater than in the no-choice condition (time point $t = 18$ s—testing for a simple difference of means using a “Bayesian t-test”^26^, the difference was $M(C-NC) = 1.70$, 95% HDI = [0.48, 2.94]). There were also instances where preference strength in the no-choice condition was greater than in the choice condition (time point $t=9$ s, $M(C-NC) = -1.25$, 95% HDI = $[-2.44, -0.00]$). Overall, the polynomial regression and direct comparisons at specific time points show that there are meaningful patterns in how preference changed over time, including oscillations within conditions and differences in mean preference and the pattern of oscillation between conditions.

Of course, the polynomial regression assumes a very specific form to the pattern of preference strength and how it should change over time. Although we used all of the degrees of freedom in the model (six coefficients for six time points), the true function relating time to mean preference strength could take a very different form than the a 5th order polynomial. To account for this, our second approach utilized a Gaussian process regression [GPR], which imposes fewer assumptions about the relationship between input (time) and output (mean preference) variables. The GPR analysis treated the measurements at different time points, within each condition, as multivariate normally distributed. In the simplest case, that could mean that outcomes within a condition and time point were all normally distributed, but the GPR also allows for correlations in preference strength across time (i.e., 3 s and 6 s should be more closely correlated than 3 s and 45 s).

The output of the GPR is a distribution over *functions* that map time points to preference judgments (outcomes). It used a squared exponential kernel and set the standard error of the mean in the GPR (the error in predicted y-values) to be equal to the largest standard error observed at any of the delays in that condition (making it as lenient as possible while still being constrained by the data). The width of the kernel determining the smoothness of the regression was set based on the within-subject covariance in preference strength between different delays. This seemed the most sensible way to fix these parameters, as it was the conservative yet still reflected the variance and covariance of preferences within the data.

The result of the GPR for Experiment 1 is shown in the left panel of Fig. 2. The mean estimated preference strength is shown as a dark red line for the choice condition and dark blue line for the no-choice condition. The shaded regions correspond to the 95% HDI on the functions mapping time to preference strength for the choice (red) and no-choice (blue) conditions. Anywhere that the dark blue line does not fall within the shaded red region and anywhere that the dark red line does not fall within the shaded blue region are instances where there is a credible difference between conditions. Aligning with the results of the Bayesian t-test comparisons and the polynomial regression, the GPR analysis shows a substantial difference between choice and no-choice conditions between approximately the 8–12 second mark (where no-choice is stronger than choice) and the 18–28 second mark (where choice is stronger than no-choice, even to the point of non-intersection).

From the results of the GPR, we can calculate effect sizes based on the overall trajectory of mean preference (dark blue/red lines in Fig. 2).The largest difference between choice and no-choice conditions was 2.80 points on the 30-point scale, translating to a medium effect size^27^ of $d = 0.44$ (*SD* of preference ratings = 8.62). The change in mean preference (maximum minus minimum) over the course of a trial was 3.50 in the choice condition ($d = 0.41$) and 5.28 in the no-choice condition ($d = 0.61$). As with the other analyses we ran, this provides further evidence of greater oscillation in the no-choice condition.

These difference were created by the oscillations in preference strength. While the GPR shows oscillating preference strength for both conditions, the greater amplitude for the oscillations of preference in no-choice condition relative to the choice condition were primarily responsible for these effects.

### Experiment 2

In Experiment 2, participants made decisions between monetary gambles rather than gift cards. These gambles had a payoff ($x) and a probability of winning the payoff (*p*), where the alternative was winning nothing ($0 with probability $$1-p$$). In this experiment, the timing of the initial choice or click (no-choice) response was varied randomly from 4 to 6 s. The timing of the preference ratings was varied randomly from 3 to 55 s across trials. For the basic analyses we present in this section, we look at the time after the first response—e.g., 10 s after initial response could be 14 s or more (4–6 s for the first prompt, plus response time for the first prompt, plus 10 s for the second response). In the generative model-based analyses in the next section, we consider the complete time course of a trial (before first prompt, choice RT, time to second prompt, and preference RT).

As in the first experiment, the first analysis we report is a Bayesian polynomial regression aimed at understanding the patterns of mean preference strength and how it changed over time. We used a fifth-order polynomial for consistency with the first study, allowing for 3–4 inversions in preference trajectory; though higher-order and lower-order polynomials yield similar results. To evaluate whether there were oscillations in preference, we examined the 3rd, 4th, and 5th order coefficients for both the choice and no-choice condition, and then compare the two conditions on these trends as well as the mean difference (intercept) between conditions.

**Table 2:** Summary of the estimated coefficients (with 95% HDIs) of the polynomial for the choice and no-choice conditions, along with the estimated difference (95% HDI) between conditions for Experiment 2. Coefficients are standardized to make the scales comparable—raw coefficients can be obtained by dividing $b_n$ by the standard deviation of the time points for the corresponding term, $SD(t^{\circ n})$ where $t = 3-55$. Differences between choice and no-choice conditions that exclude zero are highlighted in bold.

Polynomial coefficient	No choice	Choice	Difference (95% HDI)
Intercept ($b_0$)	5.65 [5.10, 6.17]	5.80 [5.33, 6.29]	${\mathbf {-0.15\,[-0.22, -0.07]}}$
Slope ($b_1$)	1.70 [0.31, 2.93]	1.39 [0.15, 2.47]	${\mathbf {0.31\,[0.14, 0.62]}}$
Quadratic ($b_2$)	$-5.44 \,[-8.71, -1.15]$	$-4.45 \,[-7.41, -0.82]$	${\mathbf {-1.01\,[-1.75, -0.18]}}$
Cubic ($b_3$)	1.83 [0.28, 3.67]	0.85 [$-0.52$, 2.44]	${\mathbf {0.98\,[0.19, 1.75] }}$
Quartic ($b_4$)	9.05 [1.47, 15.98]	8.96 [1.49, 15.83]	0.09 [$-0.03$, 0.17]
Quintic ($b_5$)	$-7.41\,[-12.53, -1.41]$	$-6.84\,[-11.84, -1.16]$	${\mathbf {-0.56\,[-0.87, -0.23]}}$

The results of this polynomial regression are shown in Table 2. Reversing the pattern in the previous study, mean preference (intercept) was higher in the choice condition ($M(b_0)=5.80$) than it was in the no-choice condition ($M(b_0)=5.65$; 95% HDI of difference $M_{diff} = [-0.22, -0.07]$). As before, the polynomial regressions were carried out within the choice and within the no-choice condition, and their values (columns 2–3 of Table 2) indicate that both conditions had some degree of oscillation. Estimates of every coefficient except the cubic component of the choice condition excluded zero, which strongly indicates the presence of oscillations in both the choice and no-choice conditions.

Furthermore, there were differences between choice and no-choice on nearly all components of the polynomial regression, beyond just a mean difference. All except the quartic component differed credibly between conditions (Table 2). We suspect that this is due to the oscillations in the two conditions being out of sync, as shown in Fig. 2, as well as the oscillations being slightly larger in the no-choice condition. This last point mimics Experiment 1, appearing to be a systematic trend across both studies. The greater amplitude indicates that choice has “stabilized” preferences, which we suggest is due to a reduction in the ontic uncertainty that happens when a person expresses their preference via choice. We cover this possibility more formally in the generative modeling section.

In addition to the polynomial regression, we also repeated the GPR analysis to gauge the evolution of preference strength over time for both conditions. For this analysis, the error of the mean GPR was set by the largest standard error of the trials within any 10-second window of delays, and the kernel was again set based on the within-subject covariance between preference ratings given to the same gamble at different time points (across different trials). The result is shown in the right panel of Fig. 2. As a reminder, the dark line indicates the pattern of mean preference, while the shaded regions lay out the variability spanned by the 95% most likely functions mapping elicitation time onto preference strength. As with the GPR for Experiment 1, we observed both instances where the mean preference strength was greater in the no-choice condition than the choice condition (blue line was above the red shaded region/red line was below the blue shaded region) and instances where mean preference strength was greater in the choice than in no-choice condition (red line was above the blue shaded region/blue line was below the red shaded region). The timing of these effects was somewhat accelerated relative to Experiment 1, likely owing to the simpler monetary gambles that had only two attributes (payoff and probability) as opposed to the four attributes in the gift cards (amount, star rating, cost, and distance). Despite this, preference strength followed a similar temporal pattern: suppression (no-choice > choice) occurred initially around 4–9 s and 24–31 s , while bolstering (choice > no-choice) occurred around 16–22 s and 34–37 s.

As in Experiment 1, we can quantify effect sizes based on the maximum differences in mean preference between and within conditions. The largest difference between choice and no-choice conditions was 0.40 on the 10-point scale, corresponding to a relatively smaller effect size of $d=0.13$ (*SD* of preference ratings = 3.22). The within-condition shifts in mean preference were slightly larger than between-condition changes, with mean preference changing by 1.07 units in the choice condition ($d = 0.33$) and 0.56 units in the no-choice condition ($d = 0.18$). These are not huge effects, but are substantive enough to be worth accounting for in our dynamic theories of preferential decision making. In the next section, we outline three dynamic theories of preference change and derive their predictions for the effects observed in our experiments.

## Generative models

The polynomial and GPR models make it clear that preference oscillates over time and that this oscillatory pattern changes depending on whether a choice is made before the preference elicitation. However, these statistical models do not explain *why* we might observe these effects. In this section, we outline and test three models of preference change. All three models share a few basic assumptions. First, they propose that a decision maker’s preference can be characterized as a state that changes over time as they gather information about the choice options. Second, this preference change can be characterized as a dynamic accumulation process, where the characteristics of the stimulus, on average, lead the decision maker to favor one option over another. Finally, preference strength ratings are a measurement of the preference state at a given point in time. They therefore follow closely in the footsteps of sequential sampling models, modifying well-established methods of modeling the dynamics of decision making and preference^1^.

A useful explanatory theory is quite a tall order: these time- and measurement-dependent properties are a serious challenge for theories that treat decisions as a read-out of a pre-existing definite preference state. Current computational models therefore struggle to parsimoniously explain how the act of making a choice can change bolstering and suppression cycles in preference evolution. In particular, most sequential sampling models based on Markovian random walk processes typically predict that, on average across trials, preference monotonically increases across time within a trial^6,8,10^. Crucially, Markov models obey the Chapman–Kolmogorov equation, which then implies that the mean trajectory of the evolution process, pooled across choices (after a choice is made), remains the same as if no choice measurement was ever elicited^12^. A similar property holds for other sequential sampling models based on other processes like independent accumulators^5,7,28^, and so they make similar predictions. We therefore must propose alternative models that are not constrained by this assumption, and so we examine quantum cognitive models^29^ that can handle differences between choice and no-choice conditions along with a hybrid variant (open system) model that incorporates advantages of both Markov and quantum models of decision making.

Before going into the details of each model, it is helpful to present in plain terms what each model is doing. The classical Markov model represents the state of the art in terms of the most common models of preferential decision making, such as multi-attribute decision field theory^1,2,8^. This model suggests that a person gathers information about the stimulus over time and changes their preference according to how much they like its attributes. In this model, a person always has a definite preference at a given point in time, and so making a choice does not change their mind about how much they like the choice options. It therefore predicts, on average, a monotonic shift in preference toward the favored option over time that does not depend on whether or not a choice is made.

The quantum and open system models add an additional cognitive mechanism to this account of preference change; namely, they assume that measuring a person’s preference state by asking them to make a choice can change their preferences. This measurement interacts with how they process information after a choice is made, resulting in more stable preferences following a choice than in absence of a choice. This additional source of ontic uncertainty ultimately allows the quantum and open system models to account for differences in the patterns of preference between choice and no-choice conditions. To understand how they generate these predictions, we examine in detail how each model represent preferences and how preference changes over time.

### Markov random walk model

To demonstrate more rigorously the properties of a Markov model for preference evolution, we applied a Markov random walk model to the data from Experiments 1 and 2. More details and methods for fitting the Markov model to the data are presented in the Methods and in the “Supplementary materials”, but the basic idea is as follows. The model consists of a set of preference levels ranging from a lower bound “definitely prefer option on left” to an upper bound “definitely prefer option on right”. The process starts with high probability near a neutral state at the center of the scale. Then there is a probability of considering an advantage for option A producing a step up the scale, or a probability of considering a disadvantage for option A producing a step down the scale, or not considering any advantage or disadvantage producing a probability to remain at the same preference level. If the process hits the top or bottom boundary, the preference stays at the bound or steps in the reverse direction. When a person is asked to make a choice followed by a later preference rating, the probability distribution is conditioned on the choice that was made, and then it evolves from that conditional distribution to generate later preference ratings. A single trial or realization of this process produces a trajectory of definite preference levels visited across time.

**Figure 3:**
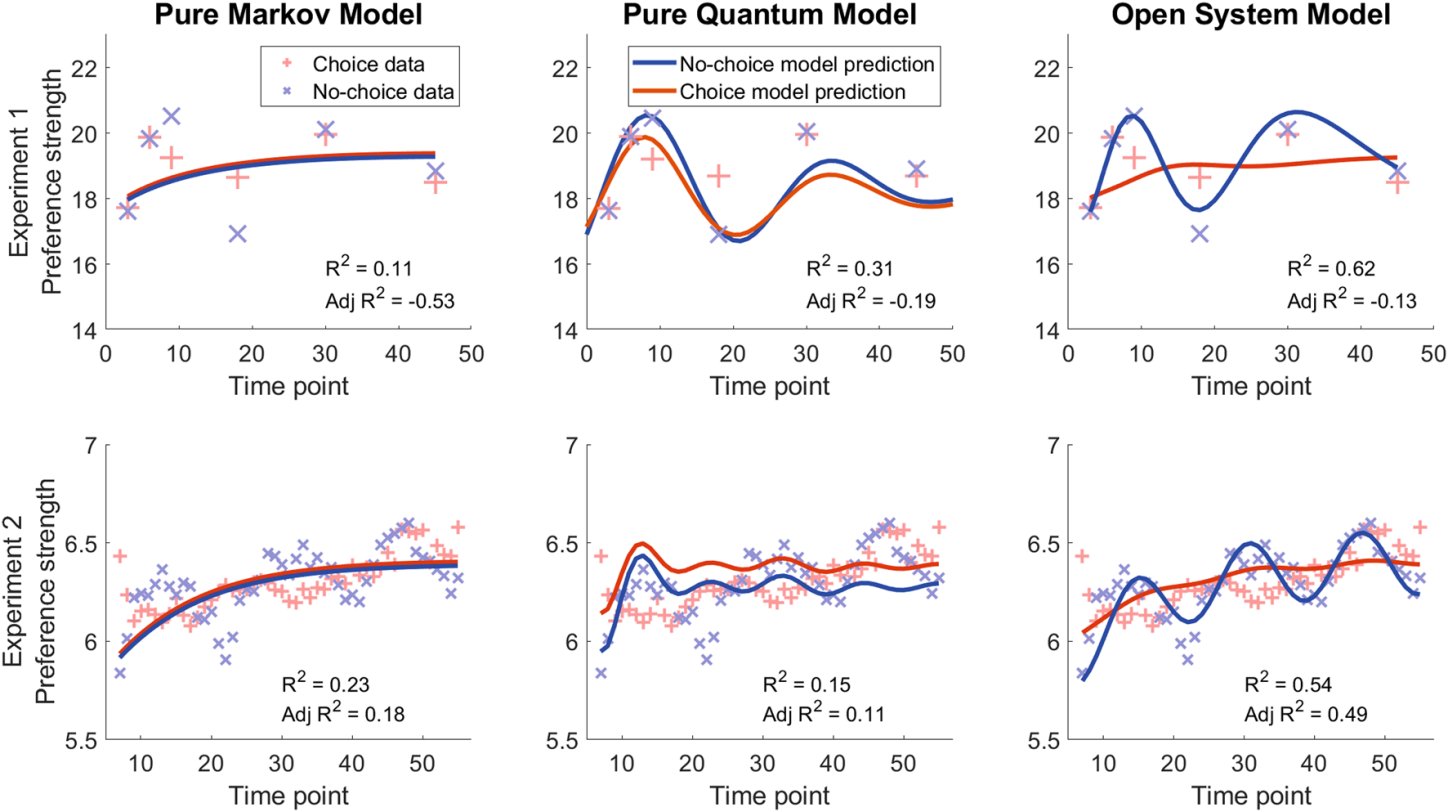
Best-fit trajectories of mean preference strength generated from the Markov (left), quantum (middle), and hybrid open system (right) models for each of the experiments. Data from the experiments are presented as red + (choice condition) or blue $\times$ (no-choice condition). For Experiment 2, the preference strength data are presented as a simple running average of responses within a $\pm 3$ s window—this pattern does not change substantially if the window is made wider or narrower, until the window is so wide that it washes out cross-time variability in mean preference. Model predictions (blue/red lines) are computed for every second after the initial response through the last time point.

The Markov random walk model was fit to the mean preference data from both experiments using three parameters: an average drift rate ($\mu _M$), a diffusion rate, ($\sigma _M$), and a decay rate that controlled how quickly people stopped paying attention or processing information related to the options ($\lambda$). Models including parameters like drift variability, start point variability, or separate non-decision times for choice and no-choice conditions have almost no noticeable effect on the qualitative pattern of model performance^12^, and so are not considered here. The results of fitting this model to the data from each experiment is shown in the left panels of Fig. 3: the Markov random walk predicts an average preference trajectory that exemplifies steady, monotonic approach to an asymptote. This is the same for both the choice and no-choice conditions, meaning that the Markov model cannot predict the diverging patterns across the choice manipulation. It thus fails to capture the two most interesting characteristics of the data: the pattern of oscillating preference and the differences in preference evolution between choice and no-choice.

Part of the issue is that Markov models often use time-invariant dynamics where a person’s preference state, averaged across the feature dimensions they could attend, moves steadily in a single direction. It is possible to equip Markov models (or other dynamic models) with time-varying dynamics via an attention switching mechanism^3,30–32^, approach-avoidance dynamics^33–35^, or different activation of goals^9^. However, this approach fails to predict (a) how many switches will occur, (b) what time the switches should occur, (c) why the timing of these switches should be systematic across individuals such that they produce systematic oscillations in the aggregate, and (d) the effect of the initial choice versus no choice condition on the subsequent dynamics of attention switching.

### Quantum walk model

Quantum walk models of decision making offer an account of both preference oscillation across time and the effect of measurement on this oscillation^12,29,36^. More details and methods for fitting the quantum walk model to the data are again presented in the “Supplementary materials”, but the basic idea is the following. Like the Markov model, the quantum model consists of a set of preference levels ranging from “definitely prefer option on left” to “definitely prefer option on right”. The process starts with an indefinite *superposition* state having amplitudes concentrated near the middle of the scale. The distribution of amplitudes can shift toward the left, right, or remain in place, depending on the advantages/disadvantages being considered. If the distribution runs up against one end of the preference scale, it stays or gets pushed back off the boundary. When a person is asked to make a choice followed by a later preference rating, the amplitude distribution is conditioned (collapsed) on the choice that was made, and then it evolves from that conditional distribution to generate later preference ratings. A single trial of this process produces a wave of amplitudes across preference levels that flows across time^14^.

It is important to note that when a choice is made both the Markov random walk and the quantum walk are conditioned (collapsed) on the choice. However, the Markov random walk predicts no effect on the marginal distribution of later preferences, whereas the quantum predicts an interference effect. Their predictions differ because the Markov process operates on probabilities, whereas the quantum process operates on amplitudes, and the latter are converted into probabilities by their squared magnitudes.

As with the Markov model, we tested the ability of a quantum walk to account for the results of our two experiments. This model had the same basic three parameters: an average drift rate ($\mu _Q$) controlling how quickly preference evolved toward one option, a diffusion rate or noise ($\sigma _Q$) describing how moment to moment variation in attention impacted preference strength, and a decay rate that controlled how quickly people stopped processing information related to the options ($\lambda$). This model was also fit to the mean patterns of preference strength ratings across the two experiments by minimizing the sum of squared error between the model prediction and average response data.

The predictions of this model are shown in the middle panels of Fig. 3. Because preference strength in the no-choice case oscillates higher than choice when preference is on the upswing, the choice creates suppression effects. Furthermore, when preference is on the downswing, the greater amplitude of no-choice oscillations result in preference being carried below preference strength for choice, creates effects where post-choice preferences are stronger than no-choice preferences.

A problem with this model is that it still does not align well with the preference patterns from the two experiments (Fig. 2). In the case of Experiment 2, it fails to capture the average drift toward the favored alternative, in effect missing the “classical” part of the accumulation process that allows preferences to settle in favor of one of the alternatives. In essence, this model picks up the ontic uncertainty associated with the preference state, explaining why asking for a choice might result in behavior diverging from no-choice and why preference might oscillate over time, but it fails to pick up the elements of epistemic uncertainty that are captured in the Markov model.

### Open system model

A general way to combine both Markov and quantum processes is achieved by using an *open system* model^37,38^. Conceptually, these models incorporate both classical epistemic uncertainty about how a person has evaluated the options alongside ontic uncertainty about what their preferences are at any given point in time, thus allowing both to be included in a complete account of preferential choice behavior. This type of model also provides a theoretically coherent way to describe a realistic neural system where information is processed in parallel and involves both cooperative and competitive interactions between neurons—i.e., one with inhibitory and excitatory connections among populations of neurons representing belief or preference strength^39^.

Formally, the open system model combines Markov and quantum walk parts into a single unified process, rather than just averaging predictions from two separate processes (see “Supplement” for details). It has six main parameters. The first two describe the classical elements of preference change: the Markov drift rate describing average preference change across people and options $\mu _M$, and the Markov diffusion rate $\sigma _M$ describing the momentary variability in preference across participants, options, and time due to fluctuations in things like attention to different attributes of the choice options. The second two parameters describe the quantum elements of preference change: the quantum drift rate $\mu _Q$ describing how quickly a person’s superposed preferences shift over time, and the quantum diffusion rate $\sigma _Q$ describing how widely their preferences become dispersed while they consider their options. The open system also includes the decay parameter, $\lambda$ indicating how quickly they stop paying attention to the options or stop evaluating new information.

The final parameter in the open system model is a mixing parameter $\alpha$ describing how much the model relies on classical or quantum elements. A value of $\alpha = 0$ indicates an entirely quantum walk model where there is a single superposition state representing ontic uncertainty, while a value of $\alpha = 1$ indicates an entirely Markov random walk model where there is a single definite preference state but epistemic uncertainty about where it is located at a given time. Naturally, this means that the quantum and Markov models are nested within the open system model and that we can find evidence for the degree of quantum-ness (tendency toward 0) or classical-ness (tendency toward 1) by examining its estimated value.

The estimates of the model parameters are presented in the “Supplementary materials”, and the fits resulting from this model are shown in the right panels of Fig. 3. The open system has no trouble accommodating the oscillation in preference and diverging patterns between choice and no-choice, resulting in bolstering and suppression effects. Unlike the quantum model, it also has no trouble accounting for the mean shift toward one of the two options over time, which is especially evident in Experiment 2. Unsurprisingly, the mean squared error for this model is much lower than the other two in both experiments, as shown in the $R^2$ values in Fig. 3. This superior performance additionally survives a penalty for additional parameters that is imposed by the adjusted R-squared metric^40^, also reported in Fig. 3. This provides support for the the open system model as the most parsimonious account of the patterns of preference change observed in our experiments.

Aside from the qualitative properties of the open system model, the values of the $\alpha$ parameter also indicate that a combination of classical and quantum elements provides the best account of the data. If one were clearly superior, $\alpha$ would tend toward 0 or 1, but instead the best estimates were $\alpha = 0.23$ for Experiment 1 and $\alpha = 0.64$ for Experiment 2. This allows the model to capture both the oscillatory pattern and the gradual mean increase in preference strength in Experiment 2. Since this mean increase is not as strong in Experiment 1, this may have resulted in a more “quantum” account (lower $\alpha$) than in Experiment 2, where there is clearly a mean shift toward stronger and stronger preferences as participants consider the options for longer periods.

## Discussion

Converging evidence from both experiments indicates that preferences systematically oscillate over time and that making a choice has a significant effect on subsequent preference ratings. In particular, choice seems to make preferences more resistant to subsequent swings, reducing the overall amplitude of oscillation in preference after the decision is made. This is reflected in the coefficients of a polynomial regression (Tables 1 and 2) as well as the result of a Gaussian process regression that makes inferences over functions rather than parameters/coefficients. Put together, these results strongly support the notion that the measurement itself (choice) has an effect on preferences by reducing the ontic uncertainty in preference: when forced to make a choice, a person must clarify their preference by ruling out preferences that favor opposing options. In the language of quantum probability, measuring a person’s preference constructs a definite preference state from an indefinite one. The resulting definite state interacts with the information processing dynamics to create diverging preference in the choice condition relative to the no-choice condition, where this definite state was never imposed.

Although oscillations in preference might strike many readers as unexpected, there appears to be some precedent for similar effects in the literature. In particular, early work in cognitive dissonance uncovered instances of apparent oscillatory preference. Walster^41^ as well as Brehm and Wicklund^42^ both found a suppression effect where ratings taken before choice/no-choice were strong than those taken after a choice. Following a suppression effect at short delays, it appeared that the more typical dissonance bolstering effect (choice > no-choice) was observed at longer delays, creating an up-and-down pattern of preference strength as a function of the time at which the judgment was elicited. The results here are at a substantially shorter timescale—0–60 s rather than 0–60 min—but appear to show many of the same oscillating properties in mean preference ratings. Later work on cognitive dissonance did not focus much on the suppression effect, perhaps because the phenomena observed in these early experiments are exceptionally difficult to explain. Isolated instances of bolstering or suppression effects can perhaps be attributed to dissonance bolstering or regret, respectively, but combining them with the oscillatory patterns in mean preference could easily have made a parsimonious account of the data elusive. As we have shown here, dynamic models of preference are seriously challenged by the combination of these phenomena. Neither purely classical Markov random walks nor pure quantum walks were able to fully capture the empirical results; only by integrating two modeling frameworks were we finally able to generate a satisfactory account of the oscillation, divergence between choice and non-choice, and average trajectories of participants’ mean preferences.

The success of this hybrid, open system account suggests that our models ought to incorporate elements of classical and quantum models. In terms of uncertainty, it appears important to include both epistemic (how did an individual participant change their preference based on the stimuli?) and ontic uncertainty (how did making a decision change their preference state?) in our representations of what participants are doing during a task. The open system model does so by jointly representing classical unpredictability of how a person will evaluate a given set of options and non-classical unpredictability of what response they will generate and how the response itself will change their preferences. This multi-layered account in many ways reflects the structure of real behavior^43^ .

In previous work, we have constructed neural oscillator models that are able to produce the operations used in quantum walk models^44^. The open system model could be similarly implementable on a classical neural network—we do not require literal quantum superpositions in the brain to implement the quantum components of the model. Instead, the open system model appears to arise naturally out of real neural systems, where information processing is highly parallelized and consists of both excitatory and inhibitory interactions between neurons. Such a system is best represented by a decoherent/hybrid model like the open system approach we have presented here^39^. Further work on the neural processes underlying decision making could explore the implementation of these networks in the brain, testing (for example) whether the act of making a choice changes the pattern of neural activity underlying preference representation and preference change.

Using a hybrid general case of both Markov and quantum accounts conferred a secondary benefit, in that it also allowed us to test for nested models. The balance of classical-ness/quantum-ness is obtained by estimating the parameter $\alpha$, which measures the degree of decoherence in the system. A fully coherent system including only ontic uncertainty, where a person maintains a superposition state over preference levels until they are asked to make a choice or judgment, would have resulted in estimates of $\alpha \rightarrow 0$. Conversely, a fully decoherent system where the decision maker always had a single definite level of preference would have resulted in estimates of $\alpha \rightarrow 1$. Neither was the case in the estimates (see Table 3), which instead indicated that a combination of these two proposals provides a better account of the data from our experiments.

## Conclusions

Research on human decision making has converged on the idea that judgments and decisions are a constructive process where preferences evolve during the selection process^3,5,7,8^ and the selection process itself exacts changes on preference^41,45–48^. Our results show that this constructive process also results in a temporal oscillation of preference. An open system model that incorporates elements of both classical and quantum dynamics provides the best available single system account of these three characteristics—evolution, oscillation, and choice-induced preference change. This open system model reveals how the interplay of different types of uncertainty—epistemic and ontic—impacts the preferences people express. Its success indicates that rich psychological mechanisms underlie the construction, maintenance, and measurement of preference, and illustrates that preference change may be an unexpectedly intricate cognitive process warranting deeper understanding.

## Methods

All methods were carried out in accordance with relevant guidelines and regulations. Experiment 1 was approved by the Michigan State University Institutional Review Board (IRB #13-1214), and Experiment 2 was approved by the Max Planck Institute for Human Development Research Ethics Board. Both experiments were deemed to involve minimal risk to participants. Prior to completing the experiments, all participants were briefed on the study procedures and completed informed consent.

The two experiments were designed to test the effect of choice on dynamically shifting preferences (Fig. 1). In both experiments, participants viewed two options (restaurant gift cards in experiment 1 and monetary gambles in experiment 2) for a few seconds, then either made a choice between them (choice condition) or an unrelated motor response (no-choice condition), then made a preference rating from “strongly prefer left” to “strongly prefer right.” The timing of this rating was varied across trials of the experiments in order to understand how participants’ preferences changed when elicited at different delays. The key outcome of interest was the mean preference following choice/no-choice: the ontic uncertainty introduced by a quantum-like process should lead to diverging oscillations in preference between choice and no-choice conditions when we compare these conditions in terms of the final preference ratings.

### Methods-Experiment 1

To examine the effects of both time and decisions on preference strength, we used a task that prompted participants to rate their preference between a pair of gift cards at different time points following a choice between those gift cards or a mouse click unspecific to the options on the screen (designed to match the motor action of making a choice but not involve the cognitive component of a decision).

Each study was run in MATLAB using Psychtoolbox-3^49^. Participants were seated in individual sound-dampening booths to complete the task. Responses (button clicks and scale selections) were gauged by tracking mouse position and left and right mouse button presses.

#### Participants

A total of 118 Michigan State University undergraduate students completed the experiment. Thirteen of these participants were dropped prior to full analysis because they each chose choice alternatives that were dominated on all attributes, included as ‘catch’ trials to identify participants that were not paying attention, on at least two out of the four occasions where this occurred. The remaining participants were all 18–30 years old (approximately 69% female, 31% male), and received class credit for participation in the study, which took no more than 1 h to complete.

#### Options

The options we used in this task were described to participants as gift cards to local restaurants. Each gift card had attributes corresponding to its value ($10–30), the star rating of the restaurant from which it came (1–5 stars), the cost of an average meal at the restaurant ($5–25), and its distance from the Michigan State University campus (0.1–10.1 miles). Most gift cards were pseudo-randomly artificially generated, with one caveat included in the generation algorithm that forced it to create gift card pairs that spanned a wide range of attribute differences (i.e. some pairs were very similar, and some had clearly dominated alternatives). One pair of gift cards in the set of options was to a real restaurant in the Lansing/East Lansing area, with the described amount of money, Yelp/star rating, distance, and an approximate meal cost calculated based on the mean cost of main course meals at that restaurant. To incentivize honest and careful performance on the task, participants received one of these real gift cards based on the choice (receiving the gift card they chose) or preference rating (receiving the gift card they rated higher) that they gave on the trial when the real gift card pair was presented.

#### Task

The task is shown in Fig. 1, upper panels. It involved making decisions and judgments about the pairs of gift cards to local restaurants. We contrasted two main conditions: a choice and a no-choice condition. In both conditions, during a trial, participants saw all four attributes for both gift cards (the amount of money on the gift card, the star rating of the restaurant, the cost of an average meal at the restaurant, and its distance from campus). In the choice condition, they would choose the item they preferred after five seconds by clicking the left or right mouse button (to choose the item on the left or right, respectively). In the no-choice condition, they would click either the left or right mouse button as they had been instructed at the beginning of that block of trials.

In both conditions, at time $t_2$, participants subsequently rated their preference between the gift cards from 0 to 30 on either side of a scale (see the rightmost panel of Fig. 3); 30 on the right would mean they strongly preferred the right-hand gift card, while 30 on the left would mean they strongly preferred the left-hand one. The timing of this preference rating was the same in the choice and no-choice conditions, allowing us to precisely map out how preferences for a chosen item change over time relative to a meaningful baseline.

#### Procedure

Participants were randomly assigned to the choice or the no-choice condition based on the session they attended, and were introduced to their specific condition of the experiment (i.e. participants in the choice condition did not see the directions for the no-choice condition). They were then seated in a sound-dampening booth and saw 48 trials of this condition of the choice/no-choice task outlined above during the experiment. These trials were blocked into groups of 12 trials, and participants in the no-choice condition would receive directions about whether to click the left or right mouse button at the beginning of each block (choice condition participants simply saw the same directions each block, telling them to click the button corresponding to the alternative they preferred). The time between $t_0$ (stimulus onset) and $t_1$ (choice or click response) was set to 5 s, at which time they were prompted to make their response with a 400 Hz auditory beep. Additionally, the time between $t_1$ and $t_2$ (preference rating) was set to 3/6/9/18/30/45 s (only one preference judgment per trial), and they were again prompted with a 400 Hz beep to make their preference response. After participants completed the task, they filled out a survey about the importance they placed on each gift card attribute and were debriefed about the aims of the study.

### Methods-Experiment 2

Study 1 aimed at identifying differences between choice and no-choice conditions at particular time points, but the findings could potentially be sensitive to the specific decision and judgment timings, the between-subjects design, or the specific options used. Experiment 2 was designed to replicate the findings from Experiment 1 in a more robust design where we used variable timing for both decisions and judgments as well as more classic gamble options commonly used in judgment and decision making experiments. It allowed us to vary the decision time and the time between choice (or the motor response in no-choice conditions) and preference judgments, and incentivize the selections more completely by paying participants according to the selections and ratings they made during the experiment.

#### Participants

The participants for Experiment 2 were 62 paid participants recruited from the subject pool at the Max Planck Institute for Human Development. Demographic information for the individuals in this study is not available, but on average, participants from this pool were approximately 25 years old (SD $\sim 3$ years), 93% students, and 50% female/50% male participants. They were paid eight euros for completing the experiment, and an additional bonus based on the outcomes of two of the gambles they chose or rated highly during the course of the experiment. For every 100 points participants received from the gambles, they received a bonus of one euro, for an average of five euros bonus across participants. The experiment took participants approximately an hour to complete.

#### Options

The options for this experiment were pairs of gambles, where each gamble consisted of an outcome in terms of points and a percentage of winning that outcome. For all gambles, participants were told that the alternative outcome (if they did not win the gamble) was 0 points.

Participants saw 20 pairs of gambles per block. Half of these (ten pairs) were repeated across blocks of trials, with their position on the screen randomized between left and right. These repeated gambles were specifically selected to be different in their attributes: one gamble would be high payoff/low probability and the other would be low payoff/high probability. The other half of the gamble pairs in each block were randomly generated, with the payoff being loosely negatively correlated with the probability of the outcome in order to avoid a high frequency of dominated gambles. There were not substantial differences in behavior between the random and repeated gambles, so we pool the results for the analyses presented below.

#### Task

An outline of the task is shown in the bottom of Fig. 1 (main text). It structure was highly similar to the gift card task, with the caveat that decision times and the time between choice and confidence were randomly sampled from a uniform distribution rather than taken at fixed delays. As in Experiment 1, each trial featured only one initial response (choice/no-choice) and one subsequent preference rating.

Each trial began when a participant clicked the fixation in the middle of the screen. The time from stimulus onset to the initial response was randomly drawn from 4 to 6 s. As before, the initial response depended on a condition manipulation of choice or no-choice. In the choice condition, participants clicked the left or right mouse button to indicate which gamble they favored. In the no-choice condition, they simply pressed the left or right mouse button according to the directions they received at the beginning of the block. As opposed to experiment 1, where the choice/no-choice manipulation was done across participants, both conditions were shown to the same participant across different blocks in experiment 2.

After a participant made their first response, the time until to the prompt for a preference rating was 2–50 s, randomly drawn for each trial. The preference scale appeared after their first response, but was greyed out until they were prompted to respond with an auditory beep. The scale then lit up and the mouse re-appeared on the screen. To make their second response, participants moved their mouse from the middle of the screen to the edge of the preference scale at the location of the response they wished to make, from 10 pink (strongly prefer gamble on the left) to 10 green (strongly prefer the gamble on the right). Their response was recorded as soon as the mouse crossed the response scale, as was the response time.

After each block, one additional gamble was presented. Participants were instructed to make their first response as in other trials, but rather than immediately moving onto the preference stage, they instead completed a survey in paper and pencil. The possible surveys were used as filler tasks so that participants could consider the gambles (or not) while performing another task for an extended period of time. The surveys are described in the “Supplementary methods” but the resulting data were not analyzed. Once the participant completed the paper survey, they continued the computer task by indicating they were finished with the mouse, and they were then asked to rate their preference for the pair of gambles they had seen before the survey. These *special gambles* were presented with a special name (e.g., “reptile”) written in color (e.g., blue) to make them memorable when they reappeared. However, preference strength between these gambles was generally weak and did not substantially differ substantially across conditions, which could be due to the intervening activity or the long delay or simple forgetting, so they are not examined in depth here.

#### Procedure

Upon arrival, participants were briefed on the task and completed informed consent before starting the experiment. There were eight total blocks in the experiment, each consisting of 20 trials (ten repeated gambles, ten random gambles). After every two blocks of trials, participants completed a special gamble and the intervening survey.

After completing all of the blocks, two of the gambles from the experiment were played so that the participant could receive the points from those trials (or not, if they did not win the gamble). One was taken from the choice trials—the chosen gamble of the pair was played. The other gamble was taken from a random pair of gambles the participant rated during the experiment. The more strongly they rated the favored gamble of the pair, the more likely it was that they got to play that gamble. Formally, their preference on the 21-point scale was mapped directly onto a 0–1 probability of the gamble being played, so a rating of $+5$ would mean a 75% chance of playing the right-hand gamble.

The computer would generate a random number to determine which gamble was played, then generate another random number to determine if they won the outcome of that gamble. For example, if the gamble was [600 points, 40%], a random number on 0–1 of 0.6 or higher meant that the participant received 600 points; if the number was less than 0.6, they did not receive any points from playing that gamble.

## Open system model

The open systems model that we used to account for the findings is summarized below. This model integrates classical/epistemic and quantum/ontic uncertainty to describe what the modeler knows about decision makers’ preference states and potential measurements. An in-depth description of these elements (density matrices, different types of uncertainty, and noise in measurement and evolution) can be found in other works^43^, but we present the essential elements involved in the model used here.

### State representation

The blend of epistemic and ontic uncertainty is captured in the state we use to describe preferences, which is a density matrix $\rho$. If there are *n* preference levels, then $\rho$ is a $n\times n$ matrix. It can be seen as a statistical distribution over possible “pure” quantum states, where there is a probability $p_j$ of being in each of *m* pure quantum states. Each pure quantum state describes a distribution of *n* (complex valued) amplitudes across the *n* preference levels for a decision maker at some time point. Mathematically, the pure quantum state is a unit length $n\times 1$ column vector denoted as $\psi _i$ ($\psi _{j}^{\dagger }$ is a row vector formed by the conjugate transpose). The density matrix is then defined as

1 $$\begin{aligned} \rho =\sum _{j}p_{j}\cdot (\psi _{j}\cdot \psi _{j}^{\dagger }) \end{aligned}$$

#### Measurement

To obtain the probability of a response, *R*, we apply a projector $P_R$. For a *n*-dimensional preference state, the projectors we use are $n \times n$ diagonal matrices with 1’s located in rows indicating the response and zeros otherwise. If the measurement is a preference rating, then the projector $P_j$ would have a one located in the row corresponding to preference level *j*. If the measurement is a binary choice between options A versus B, then a projector for choosing A, $P_A$, would be used with 1’s located across the range of rows for which the preference level favors option A over B. The probability of obtaining a particular response *R* and represented by the projector $P_R$, is determined by $Pr(R) =$ Tr $(P_R \cdot \rho \cdot P_R^\dagger )$, where Tr is the “trace” operator that computes the sum of the entries along the main diagonal. The trace of the density matrix $\rho$ must be 1, meaning that the probabilities of obtaining all of the different (mutually exclusive) measurements sum to one.

#### Dynamics

Like the density matrix, the dynamics have both classical and quantum parts as well. The density matrix evolves across time according to the following open system model:

2 $$\begin{aligned} \frac{d}{dt} \rho (t)&=-i\cdot \left( 1-\alpha \right) \cdot \left[ H,\rho \right] +\alpha \cdot {\mathscr {L}}\left( \rho \right) ,\nonumber \\ {\mathscr {L}}\left( \rho \right)&=\sum \gamma _{ij}\cdot \left( L_{ij}\cdot \rho \cdot L_{ij}^{\dagger }-\frac{1}{2}\left\{ L_{ij}^{\dagger }\cdot L_{ij},\rho \right\} \right) ,\nonumber \\ \left[ H,\rho \right]&=H\cdot \rho -\rho \cdot H,\nonumber \\ \left\{ \left( L_{ij}^{\dagger }\cdot L_{ij}\right) ,\rho \right\}&=\left( L_{ij}^{\dagger }\cdot L_{ij}\right) \cdot \rho +\rho \cdot \left( L_{ij}^{\dagger }\cdot L_{ij}\right) \end{aligned}$$

where $L_{ij}$ is a $n\times n$ matrix with a one in cell *i*, *j* and zeros otherwise. The first part, $\left[ H,\rho \right]$, of the sum in Eq. (2) represents von Neumann dynamics produced by a pure quantum system, and the second part, ${\mathscr {L}}\left( \rho \right) ,$ of the sum in Eq. (2) represents the Lindblad dynamics of a Markov system. The parameter, $h_{ij}$, in the $n\times n$ Hermitian matrix $H=H^{\dagger }$ determines the rate of change in the transition amplitudes to preference level *i* from another preference level $j\in S$. The parameters $\gamma _{ij},i,j=1,\ldots ,n$ which form a matrix, determine the rate of change in the probability of transiting to preference level *i* from another preference level *j*. The open system model integrates quantum and Markov systems. The system starts out operating in the quantum regime to produce a coherent density matrix with non-zero off-diagonal cells, but later the system ends in the Markov regime to produce a decoherent density matrix with zero off-diagonal cells. The parameter $0\le \alpha \le 1$ determines the weight of the contribution of each process. A higher value of $\alpha$ will therefore influence the components of the state more in terms of classical epistemic uncertainty, while a lower value of $\alpha$ will push it toward pure ontic uncertainty. At the extremes, $\alpha = 0$ will result in a pure quantum process and $\alpha = 1$ will result in a pure Markov process. These two matrices *H* and *K* are crafted from a *Hamiltonian* *H* and an *intensity matrix* *K*, respectively. We first construct the Hamiltonian and then shift focus to the intensity matrix.

The Hamiltonian describes the quantum component of the continuous time dynamics in terms of a drift $\mu _Q$ that controls the rate at which quantum component of the state (superposition state) shifts toward the favored alternative, and diffusion rate $\sigma _Q$ that controls the diffusion of amplitudes, inducing uncertainty over different preference levels. Supposing that we have a set of *n* latent preference levels, the Hamiltonian describing the dynamics of the quantum part of the state is specified as the tridiagonal Hermitian matrix

$$\begin{aligned} H=\left[ \begin{array}{lllll} \mu _Q(1) &{} \sigma _Q &{} 0 &{} &{} 0\\ \sigma _Q &{} \mu _Q(2) &{} \ddots \\ 0 &{} \sigma _Q &{} \ddots &{} \sigma _Q &{} 0\\ &{} &{} \ddots &{} \mu _Q(n-1) &{} \sigma _Q\\ 0 &{} &{} 0 &{} \sigma _Q &{} \mu _Q(n) \end{array}\right] \end{aligned}$$

The main diagonal $\mu _Q(j)$ in this case is a linear function of *j* (though it can be quadratic in other applications). In our application, it is simply set as $\mu _Q(j) = j \cdot \mu _Q$—the intercept does not matter, only the slope. The resulting potential “pushes” a person’s preference toward the higher-index preference levels (if $\mu _Q$ is positive) or lower-index preference levels (if $\mu _Q$ is negative). We treat higher indices as more extreme preference levels, so that higher values of $\mu _Q$ will result in stronger observed preferences when it comes to the decision maker’s responses on the preference scale (Fig. 1 (main text)).

The classical Markov component of the continuous time dynamics are specified by a tridiagonal intensity matrix *K*, which is also specified using a drift rate $\mu _M$ and a diffusion rate $\sigma _M$.

$$\begin{aligned} K=\left[ \begin{array}{lllll} (-\sigma _M - \mu _M) &{} (\sigma _M - \mu _M) &{} 0 &{} 0 &{} 0\\ (\sigma _M + \mu _M) &{} -2 \sigma _M &{} \ddots \\ 0 &{} \sigma _M + \mu _M &{} \ddots &{} \sigma _M - \mu _M &{} 0\\ 0 &{} &{} \ddots &{} -2 \sigma _M &{} (\sigma _M - \mu _M)\\ 0 &{} 0 &{} 0 &{} (\sigma _M + \mu _M) &{} (-\sigma _M + \mu _M) \end{array}\right] \end{aligned}$$

As before, the drift rate $\mu _M$ will push the preference state toward the higher indices (lower rows in the matrix), indicating greater support for the favored option. The diffusion rate $\sigma _M$ will result in more widely dispersed preferences, indicating inconsistent effects of options within or across participants.

#### Synthesis

We still need to put the pieces together to understand how the model predicts preference ratings at different time points following choice or no choice. A decision maker’s state at time 0, right as the option appears and before they have considered any information about it, is assumed to be unbiased with respect to the two choices. This is represented by $\rho (t_{0})=\psi _{0}\cdot \psi _{0}^{\dagger }$, where $\psi _{0}$ has amplitudes equal to the square root of a normalized truncated normal distribution with a mean at the middle preference level, $mid=(n+1)/2$, and a standard deviation $s_v$. As we suggest below, the starting point variability/standard deviation had essentially no effect on mean preference trajectories so it was set to half the width of the scale.

Preferences then evolve according to the open system dynamic process during the length of time between $t_0$ and $t_1$. In Experiment 1, this is $t = t_1 - t_0 = 5$ s, whereas in Experiment 2 it is uniformly drawn between 4 and 6 s. At time $t_1$, in the choice condition, a measurement of the state is taken. The likelihood of selecting the option on the right is $Pr_{t1}(R) = Tr(P_R \cdot \rho _{t_1} \cdot P_R^\dagger )$, where $P_R$ is a matrix with 1s along the main diagonal for states above *mid* and 0s everywhere else (except at *mid*). Conversely, the likelihood of selecting the option on the left is $Pr_{t1}(L) = Tr(P_L \cdot \rho _{t_1} \cdot P_L^ \dagger )$, where $P_L$ is a matrix with 1s along the main diagonal for states below *mid* and 0s everywhere else (except at *mid*). At *mid* the value is $\sqrt{(}\frac{1}{2})$.

The place where the choice and no-choice conditions diverge is described next. Decision makers who have made a choice change to reduced states $\rho _{t_1,R} = P_R \rho _{t_1}/Pr_{t1}(R)$ or $\rho _{t_1,L} = P_L \rho _{t_1}/Pr_{t1}(L)$, while decision makers who were prompted for just a click (no-choice) remain in state $\rho _{t_1}$. This applies to all models, that is when $\alpha =0$, and when $\alpha =1$, and when $\alpha$ is a free parameter. As a result, the subsequent evolution of the state can diverge between the choice and no-choice conditions.

The second part of the preference dynamics are influenced by the rate of decay $\lambda$, which determines how quickly decision makers stop paying attention to new information. With delays of $t_2 - t_1 = 3$–50 s (across experiments), some participants will naturally lose interest and therefore their “dynamics” will cease. Thus, the dynamics between the first (choice/no-choice) and second (preference rating) response will depend on the the value of $\lambda$ which is used to rescale the time scale according to

3 $$\begin{aligned} \tau _2= t_2 \cdot \exp (-\lambda [t_2-t_1]) \end{aligned}$$

The exponentially decreasing factor $\exp (-\lambda [t_2-t_1])$ corresponds to a simple assumption that participants “drop out” and stop processing new information at a constant rate. The probability of observing preference level *j* at time $t_2$ is then equal to $Pr(j) = Tr(P_j \rho _{\tau _2})$.

We only consider the *strength* of preference ratings (i.e., their absolute difference from the midpoint), and so we take the absolute value of the difference between the observed preference level and the midpoint of the preference scale (*mid*). This is then mapped onto a mean preference strength $S = Tr(S_{range} \cdot \rho _{t_2})$. Here, $S_{range}$ is a diagonal matrix with the rating corresponding to a preference level specified along the main diagonal. We use a simple logistic mapping, so that the distance between the observed state and the midpoint of the preference state space is mapped onto a number between 0 and 1, then turned into a rating on the scale:

4 $$\begin{aligned} \forall j, S_{j,j} = S_{max} \cdot \frac{1}{1 + \exp (-|j - mid|)} \end{aligned}$$

The value of $S_{max}$ was given by the scale, so that $S_{max} = 30$ for Experiment 1 and $S_{max} = 10$ for Experiment 2. More complex mappings could also be considered in future work, but this appeared sufficient at present.

Taken together, this gave the expected preference strength rating distributions for choice and no choice conditions, where the distribution from the choice condition was weighted by the probability of each response, $Pr_{t_2}( S_j) = Pr_{t1}(R) \cdot Pr_{t_2}(S_j|R) + Pr_{t1}(L) \cdot Pr_{t_2}(S_j|L)$. The results of this type of process are shown in Fig. 3 (main text).

### Application to the data

The first issue that needed to be addressed was the dimension *n* of the quantum state. Since we were only fitting the mean trajectory of preference states, the number of dimensions had little effect on the overall performance (a larger number of dimensions just means that the drift and diffusion parameters would need to be adjusted to compensate for the larger number of states the process would traverse). For the results in the main text, we used a 21-dimensional representation, which was large enough to ensure reasonable precision but small enough to be computationally tractable. One can get almost identical results with 5 states or 61 states, so the number of dimensions appears to be mainly a scaling factor that can be set arbitrarily when fitting the means.

There are at least two ways to solve the matrix differential Eq. (2). One is to use a numerical differential equation solver. Another method (see reference^25^) is to vectorize density matrix into a super-vector and use the exponential solution to a super-vector differential equation. We used the latter methods.

The models were all fit by minimizing the squared difference between the true observed mean preference strength and the preferences predicted by the model. For the open system model, we allowed all seven parameters to vary freely: drift on the quantum part of the model ($\mu _Q$), drift on the classical part of the model ($\mu _M$), diffusion in the quantum part of the model ($\sigma _Q$), diffusion in the classical part of the model ($\sigma _M$), the Markov-quantum trade-off parameter ($\alpha$), start point variability ($s_v$), and the rate of decay in participants’ attention following their first response ($\lambda$). Start point variability was found to have essentially no effect on mean preference trajectories, so it was fixed at one half of the width of the response scale.

Both the Markov model and the quantum model can be fit as special cases of the open system model. For the pure Markov model, $\alpha$ was set to 1, which exactly produces a continuous time Markov process. Conversely, for the pure quantum model, $\alpha$ was set to 0, which exactly produces a continuous time quantum process.

To fit the remaining parameters, 1000 random starting points were drawn and fed into a gradient descent optimization procedure (fminsearch in MATLAB). The parameters producing the best (lowest squared error) estimate of preferences were kept and used to construct the trajectories shown in Fig. 3 (main text). The exact values of all of these parameters are shown in Table 3.

**Table 3:** Parameter estimates for the open system model presented here (right panels of Fig. 3).

Open System parameter	Experiment 1	Experiment 2
Drift (quantum)	226.8	633.8
Diffusion (quantum)	10.6	17.9
Drift (Markov)	28.5	18.3
Diffusion (Markov)	122.5	25.1
Alpha	0.285	0.641
Decay	0.014	0.458

Code for computing and fitting the models, along with the data from the two experiments, are provided on the Open Science Framework at https://osf.io/vknj6/.

## Supplementary information

Supplementary Information.
