# Supplementary material for: Temporal oscillations in preference strength provide evidence for an open system model of constructed preference
Source: Sci Rep. 2021 Apr 14;11:8169. doi: 10.1038/s41598-021-87659-0 (PMC8046775; doi:10.1038/s41598-021-87659-0)
Supplement: Supplementary file 1 — Supplementary Information. [file 41598_2021_87659_MOESM1_ESM.pdf]

Supplementary material accompanying "Temporal oscillations in preference strength provide evidence for an open system model of constructed preference"

Peter D. Kvam
University of Florida

Jerome R. Busemeyer
Indiana University

Timothy J. Pleskac
University of Kansas

Methods - Experiment 1

To examine the effects of both time and decisions on preference strength, we used a task that prompted participants to rate their preference between a pair of gift cards at different time points following a choice between those gift cards or a mouse click unspecific to the options on the screen (designed to match the motor action of making a choice but not involve the cognitive component of a decision).

Each study was run in MATLAB using Psychtoolbox-3 (Kleiner et al., 2007). Participants were seated in individual sound-dampening booths to complete the task. Responses (button clicks and scale selections) were gauged by tracking mouse position and left and right mouse button presses.

Participants. A total of 118 Michigan State University undergraduate students completed the experiment. Thirteen of these participants were dropped prior to full analysis because they each chose choice alternatives that were dominated on all attributes, included as 'catch' trials to identify participants that were not paying attention, on at least 2 out of the 4 occasions where this occurred. The remaining participants were all 18-30 years old (approximately 69% female, 31% male), and received class credit for participation in the study, which took no more than 1 hour to complete.

Options. The options we used in this task were described to participants as gift cards to local restaurants. Each gift card had attributes corresponding to its value ($10-30), the star rating of the restaurant from which it came (1-5 stars), the cost of an average meal at the restaurant ($5-25), and its distance from the Michigan State University campus (0.1 – 10.1 miles). Most gift cards were pseudo-randomly artificially generated, with one caveat included in the generation algorithm that forced it to create gift card pairs that spanned a wide range of attribute differences (i.e. some pairs were very similar, and some had clearly dominated alternatives). One pair of gift cards in the set of options was to a real restaurant in the Lansing / East Lansing area, with the described amount of money, Yelp / star rating, distance, and an approximate meal cost calculated based on the mean cost

of main course meals at that restaurant. To incentivize honest and careful performance on the task, participants received one of these real gift cards based on the choice (receiving the gift card they chose) or preference rating (receiving the gift card they rated higher) that they gave on the trial when the real gift card pair was presented.

Task. The task is shown in Figure 1 (main text), upper panels. It involved making decisions and judgments about the pairs of gift cards to local restaurants. We contrasted two main conditions: a choice and a no–choice condition. In both conditions, during a trial, participants saw all four attributes for both gift cards (the amount of money on the gift card, the star rating of the restaurant, the cost of an average meal at the restaurant, and its distance from campus). In the choice condition, they would choose the item they preferred after five seconds by clicking the left or right mouse button (to choose the item on the left or right, respectively). In the no–choice condition, they would click either the left or right mouse button as they had been instructed at the beginning of that block of trials.

In both conditions, at time t_2, participants subsequently rated their preference between the gift cards from 0 to 30 on either side of a scale (see the rightmost panel of Figure 3); 30 on the right would mean they strongly preferred the right-hand gift card, while 30 on the left would mean they strongly preferred the left-hand one. The timing of this preference rating was the same in the choice and no–choice conditions, allowing us to avoid the issues with pre-post ratings of the free choice paradigm as well as more precisely map out how preferences for a chosen item change over time relative to a meaningful baseline.

Procedure. Participants were randomly assigned to the choice or the no–choice condition based on the session they attended, and were introduced to their specific condition of the experiment (i.e. participants in the choice condition did not see the directions for the no–choice condition). They were then seated in a sound-dampening booth and saw 48 trials of this condition of the choice / no–choice task outlined above during the experiment. These trials were blocked into groups of 12 trials, and participants in the no–choice condition would receive directions about whether to click the left or right mouse button at the beginning of each block (choice condition participants simply saw the same directions each block, telling them to click the button corresponding to the alternative they preferred). The time between t_0 (stimulus onset) and t_1 (choice or click response) was set to 5 seconds, at which time they were prompted to make their response with a 400Hz auditory beep. Additionally, the time between t_1 and t_2 (preference rating) was set to 3 / 6 / 9 / 18 / 30 / 45 seconds (only one preference judgment per trial), and they were again prompted with a 400Hz beep to make their preference response. After participants completed the task, they filled out a survey about the importance they placed on each gift card attribute and were debriefed about the aims of the study.

Methods - Experiment 2

Study 1 aimed at identifying differences between choice and no–choice conditions at particular time points, but the findings could potentially be sensitive to the specific decision and judgment timings, the between-subjects design, or the specific options used. Experiment 2 was designed to replicate the findings from Experiment 1 in a more robust design where we used variable timing for both decisions and judgments as well as more classic gamble options commonly used in judgment and decision making experiments. It allowed us to vary the decision time and the time between choice (or the motor response in no–choice

conditions) and preference judgments, and incentivize the selections more completely by paying participants according to the selections and ratings they made during the experiment.

Participants. The participants for Experiment 2 were 62 paid participants recruited from the subject pool at the Max Planck Institute for Human Development. Demographic information for the individuals in this study is not available, but on average, participants from this pool were approximately 25 years old (SD $\sim$3 years), 93% students, and 50% female / 50% male participants. They were paid eight euros for completing the experiment, and an additional bonus based on the outcomes of two of the gambles they chose or rated highly during the course of the experiment. For every 100 points participants received from the gambles, they received a bonus of one euro, for an average of five euros bonus across participants. The experiment took participants approximately an hour to complete.

Options. The options for this experiment were pairs of gambles, where each gamble consisted of an outcome in terms of points and a percentage of winning that outcome. For all gambles, participants were told that the alternative outcome (if they didn't win the gamble) was 0 points.

Participants saw 20 pairs of gambles per block. Half of these (10 pairs) were repeated across blocks of trials, with their position on the screen randomized between left and right. These repeated gambles were specifically selected to be different in their attributes: one gamble would be high payoff / low probability and the other would be low payoff / high probability. The other half of the gamble pairs in each block were randomly generated, with the payoff being loosely negatively correlated with the probability of the outcome in order to avoid a high frequency of dominated gambles. There were not substantial differences in behavior between the random and repeated gambles, so we pool the results for the analyses presented below.

Task. An outline of the task is shown in the bottom of Figure 1 (main text). It structure was highly similar to the gift card task, with the caveat that decision times and the time between choice and confidence were randomly sampled from a uniform distribution rather than taken at fixed delays. As in Experiment 1, each trial featured only one initial response (choice / no–choice) and one subsequent preference rating.

Each trial began when a participant clicked the fixation in the middle of the screen. The time from stimulus onset to the initial response was randomly drawn from 4-6 seconds. As before, the initial response depended on a condition manipulation of choice or no–choice. In the choice condition, participants clicked the left or right mouse button to indicate which gamble they favored. In the no–choice condition, they simply pressed the left or right mouse button according to the directions they received at the beginning of the block. As opposed to experiment 1, where the choice / no-choice manipulation was done across participants, both conditions were shown to the same participant across different blocks in experiment 2.

After a participant made their first response, the time until to the prompt for a preference rating was 2-50 seconds, randomly drawn for each trial. The preference scale appeared after their first response, but was greyed out until they were prompted to respond with an auditory beep. The scale then lit up and the mouse re-appeared on the screen. To make their second response, participants moved their mouse from the middle of the screen to the edge of the preference scale at the location of the response they wished to make, from 10 pink (strongly prefer gamble on the left) to 10 green (strongly prefer the gamble on the

right). Their response was recorded as soon as the mouse crossed the response scale, as was the response time.

After each block, one additional gamble was presented. Participants were instructed to make their first response as in other trials, but rather than immediately moving onto the preference stage, they instead completed a survey in paper and pencil. The possible surveys were used as filler tasks so that participants could consider the gambles (or not) while performing another task for an extended period of time. The surveys are described in the supplementary methods but the resulting data were not analyzed. Once the participant completed the paper survey, they continued the computer task by indicating they were finished with the mouse, and they were then asked to rate their preference for the pair of gambles they had seen before the survey. These *special gambles* were presented with a special name (e.g., “reptile”) written in color (e.g., blue) to make them memorable when they reappeared. However, preference strength between these gambles was generally weak and did not substantially differ substantially across conditions, which could be due to the intervening activity or the long delay or simple forgetting, so they are not examined in depth here.

Procedure. Upon arrival, participants were briefed on the task and completed informed consent before starting the experiment. There were 8 total blocks in the experiment, each consisting of 20 trials (10 repeated gambles, 10 random gambles). After every 2 blocks of trials, participants completed a special gamble and the intervening survey.

After completing all of the blocks, 2 of the gambles from the experiment were played so that the participant could receive the points from those trials (or not, if they did not win the gamble). One was taken from the choice trials – the chosen gamble of the pair was played. The other gamble was taken from a random pair of gambles the participant rated during the experiment. The more strongly they rated the favored gamble of the pair, the more likely it was that they got to play that gamble. Formally, their preference on the 21-point scale was mapped directly onto a 0-1 probability of the gamble being played, so a rating of +5 would mean a 75% chance of playing the right-hand gamble.

The computer would generate a random number to determine which gamble was played, then generate another random number to determine if they won the outcome of that gamble. For example, if the gamble was [600 points, 40%], a random number on 0-1 of 0.6 or higher meant that the participant received 600 points; if the number was less than 0.6, they did not receive any points from playing that gamble.

Analyses

The data from these experiments were analyzed in two ways for the main statistical analyses presented in the main text: a polynomial regression and a Gaussian process regression. The polynomial regression estimated how mean preference strength (difference between the preference rating on a given trial and zero) changed as a function of the time t at which preference was elicited. Because there were six discrete time points in Experiment 1, we estimated a 5th order polynomial to account for how preference changed across these time points. This allows us to fully capture the mean preference data y in each condition using six coefficients b_0, b_1, b_2, b_3, b_4, b_5:

$$y = b_0 + b_1 t + b_2 t^2 + b_3 t^3 + b_4 t^4 + b_5 t^5 \quad (1)$$

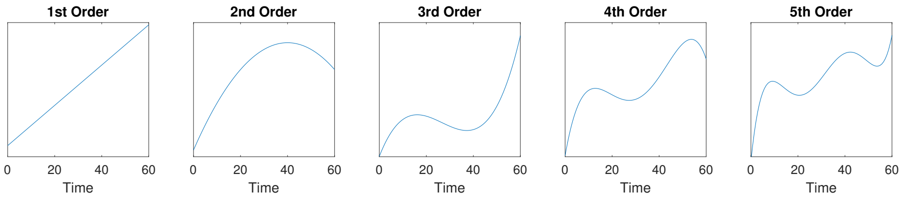

Figure 1. Patterns of data that can be captured by different polynomials. To allow for all possible patterns of oscillation, we used a 5th order polynomial to describe the preference dynamics in Experiment 1.

This allowed us to capture as many inversions (reverses in the slope of mean preference over time) as there could be in the data. This is illustrated in Figure 1. With six data points, we can capture a pattern of preference ratings that reverses trajectory four times – the first two points set the initial "direction" of mean preference change, and the third through sixth points can each reverse this direction. As a result, we could expect oscillations to appear in the third, fourth, or fifth order coefficient of the model in Equation 1.

To understand oscillations, we can therefore look at the estimates of the third, fourth, and fifth order coefficients b_3, b_4, or b_5. If preference change is monotonic, we would typically not expect to see any effect in these coefficients (although the third-order coefficient could result from a logistic growth curve). Therefore, the fact that several of these coefficients showed credibly nonzero effects, reported in Table 1 of the main text, indicates that there is oscillation in mean preference with respect to time.

We can also use these polynomial coefficients to contrast the choice and no-choice conditions. A mean difference in preference strength would show up as a difference between the b_0 estimates between conditions, a difference in the slope of preference change would show up as a difference between the b_1 coefficients, and so on. A difference in the amplitude or phase of oscillation between conditions would show up in the third, fourth, or fifth order coefficients. As we show in the main text, such differences are substantiated in differences in b_3 and b_5 between choice and no-choice conditions in both experiments.

Note that it would be possible to analyze Experiment 2 with a higher-order polynomial. Because time was continuously randomly drawn on each trial, there are as many time points as there are data. Therefore, we are not limited to the fifth order polynomial used in Experiment 1. However, we opted to perform the same polynomial regression in Experiment 2 for two reasons. First, it avoided using up too many degrees of freedom and artificially inflating the number of credible coefficients by overfitting the data. Limiting ourselves to a fifth order model constrains the number of coefficients and thus the Type I error rate in detecting effects that are not there.

Additionally, matching the polynomials between experiments allows us to evaluate the extent to which they align with one another. As shown in Tables 1 and 2, the cubic and quartic coefficients are both positive (for both choice and no-choice conditions) and the quintic coefficients are negative (again for both choice and no-choice conditions). This indicates that the same overall pattern of oscillation appears in both experiments. Furthermore, the differences between choice and no-choice conditions in the third and fifth order coefficients (b_3 and b_5, rightmost columns of Tables 1 and 2) are positive and negative in both experiments, respectively. This indicates that the differences in oscillation between choice

and no-choice conditions appear to be consistent, apparently favoring greater amplitude oscillations in the no-choice condition than in the choice condition.

Finally, for those readers more comfortable with classical statistical tests of these results, we also examined the polynomial analysis using classical statistical tests. We performed a multivariate general linear model analysis for repeated measures to test the orthogonal contrasts for polynomial trend components and their interaction with the choice and no choice condition. In experiment 1, the standardized effect size of the fifth order trend (oscillatory component) was 0.95 ($p < .001$). In experiment 2, the standardized effect size of this trend was 0.39 ($p = .03$). Because these were orthogonal coefficients, they quantified the degree to which each trend contributed *unique* variance, i.e., these effect sizes / p-values quantify the degree to which predictions are improved by adding a $5th$ order coefficient to a 4th order model. In both experiments, the 5th-order trends were significant, meaning that the polynomial regression supported the presence of oscillations within both the choice and no-choice conditions. Furthermore, the (again orthogonal) effect of the condition manipulation was significant (explained additional variance beyond the model with no difference between conditions), indicating a difference between conditions. To summarize, for Experiment 1, the F test of the 5th order trend component interaction with condition produced $F(1,103) = 8.3$, $p = 0.005$ and effect size = 0.56; for Experiment 2, the F test of the 5th order trend component interaction with condition produced $F(1,61) = 6.00$, $p = .017$, effect size = 0.31. These lined up well with the results of the Bayesian analysis, which suggested a medium effect size and difference between conditions in the oscillations in the first experiment and a slightly smaller but still significant effect in the second experiment.

References

Kleiner, M., Brainard, D., Pelli, D., Ingling, A., Murray, R., & Broussard, C. (2007). What's new in psychtoolbox-3. *Perception*, *36*(14), 1.
